# Unilateral Diffuse Alveolar Hemorrhage as an Initial Presentation of Microscopic Polyangiitis

**DOI:** 10.7759/cureus.46813

**Published:** 2023-10-10

**Authors:** Tapas Ranjan Behera, Amninder Kaur, Satyajit Acharya, Prabhat Mallick, Alisha Sahu

**Affiliations:** 1 Nephrology, Institute of Medical Sciences (IMS) and SUM Hospital, Bhubaneswar, IND; 2 Nephrology, SUM Ultimate Medicare, Bhubaneswar, IND; 3 Pulmonology, SUM Ultimate Medicare, Bhubaneswar, IND; 4 Anesthesiology, Institute of Medical Sciences (IMS) and SUM Hospital, Bhubaneswar, IND

**Keywords:** plasmapheresis, microscopic polyangiitis, glomerulonephritis, diffuse alveolar hemorrhage, anca vasculitis

## Abstract

Diffuse alveolar hemorrhage (DAH) is a life-threatening condition due to widespread damage to small pulmonary vessels commonly caused by systemic vasculitis. Alveolar involvement is typically multi-lobar and bilateral. It frequently presents as bilateral diffuse airspace opacities on chest imaging. Unilateral DAH is rare. Patients presenting with hemoptysis, anemia, hypoxemia, progressive dyspnea, and opacities on chest imaging should be evaluated for systemic vasculitis such as antineutrophilic cytoplasmic antibody (ANCA) vasculitis. We report the case of a 23-year-old female who presented with hemoptysis, severe dyspnea, hypoxemia, anemia, and oliguria. The laboratory exam results showed the patient to be p-ANCA positive, which suggests a diagnosis of microscopic polyangiitis. Chest X-ray showed unilateral airspace opacities, and DAH was confirmed by hemosiderin-laden macrophages on bronchoalveolar fluid histopathological examination. After treatment with plasmapheresis, intravenous methylprednisolone pulse, and cyclophosphamide, the patient’s symptoms and radiographic findings improved.

## Introduction

Pulmonary renal syndromes are rare clinical entities that usually present with glomerulonephritis (GN) and diffuse alveolar hemorrhage (DAH). DAH is a rare but life-threatening condition that usually presents with hemoptysis, anemia, and bilateral/multi-lobar lung infiltration [1]. However, it can present as a non-specific finding as unilateral lung infiltration, making its early diagnosis difficult. DAH is frequently caused by antineutrophil cytoplasmic antibody (ANCA)-associated vasculitis (AAV). AAV is an inflammatory disease of small blood vessels. Early diagnosis and immediate treatment will lead to better outcomes in these patients. We report here a case of a patient with microscopic polyangiitis (MPA) with unilateral DAH, which was successfully managed with plasmapheresis and immunosuppression.

## Case presentation

**Figure 1:**
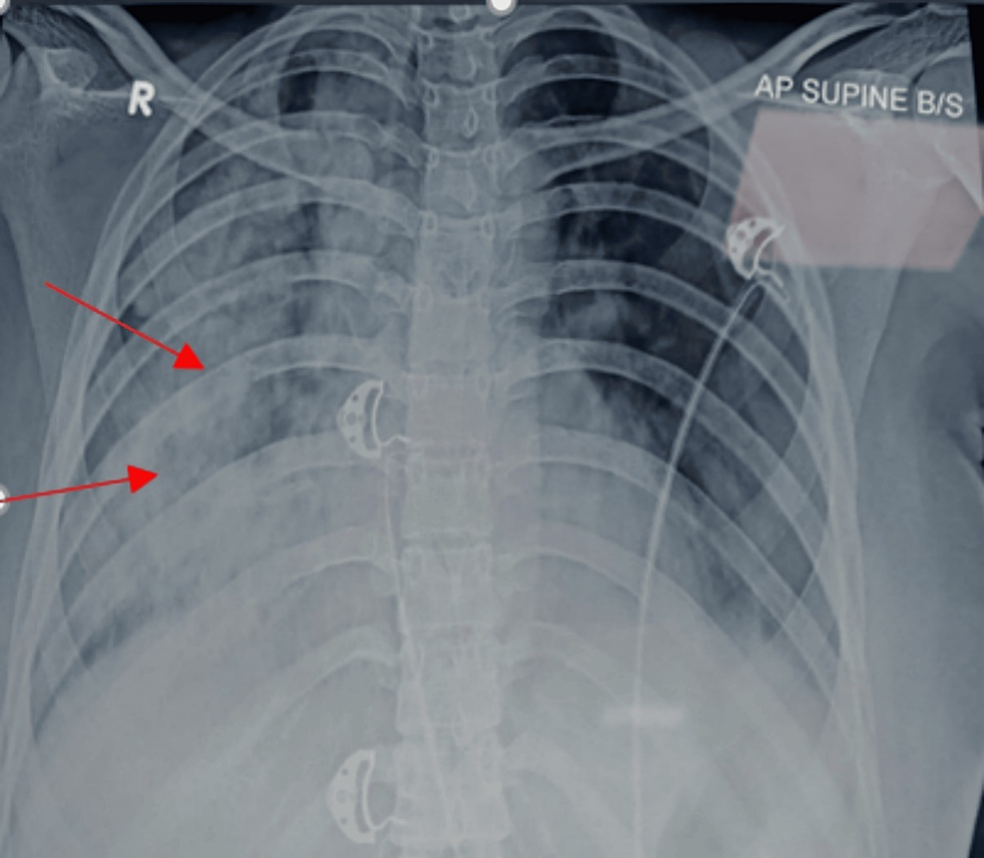
Chest X-ray showing unilateral lung infiltrates (red arrows)

**Figure 2:**
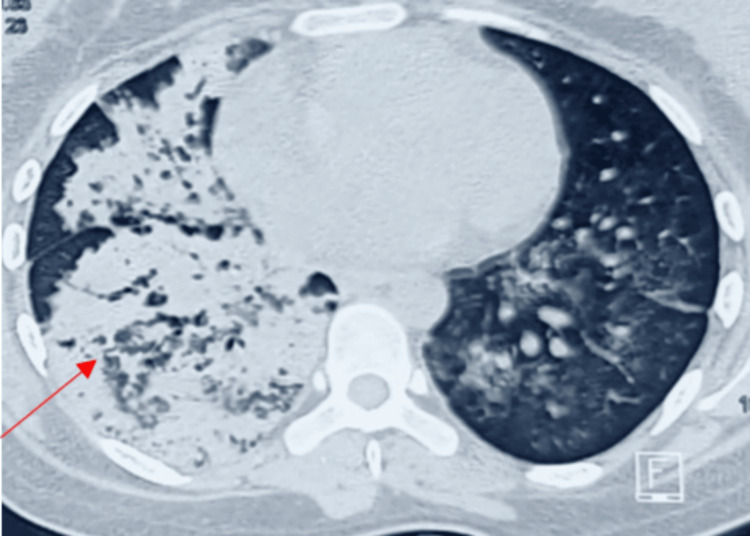
Chest high-resolution computed tomography (HRCT) showing right unilateral diffuse alveolar opacities (red arrow)

A 23-year-old female presented to the outpatient department with complaints of hemoptysis, dyspnea, and generalized weakness for seven days. The patient had no significant past medical history such as hypertension, diabetes mellitus, or hypothyroidism. She was not on any other medications. She denied having any travel history. Her baseline serum creatinine and hemoglobin levels were not available. On initial evaluation, blood pressure was 130/90 mmHg, pulse rate was 98 beats per minute, respiratory rate was 20 breaths per minute, temperature was 36.5°C, and saturation was 94% on room air. Initial laboratory findings were as follows: hemoglobin at 5.5 gm/dL, leukocyte count at 10,070 cells/cumm, platelet count at 127,000 cells/cumm, urea at 128 mg/dL, serum creatinine at 7.95 mg/dL, and potassium at 5.5 mEq/L. Liver function test levels were within normal limits. The urine dipstick test results showed 3+ protein and 2+ blood, and microscopy showed RBC 119/hpf. Ultrasonography of the abdomen showed a 9.1 x 4.1 cm right kidney and a 9.9 x 4.3 cm left kidney with a mild increase in echogenicity and no evidence of stone or hydroureteronephrosis. Chest radiography showed unilateral lung infiltrates (Figure 1) and high-resolution computed tomography (HRCT) showed unilateral diffuse alveolar opacities (Figure 2) in the entire lung field. Radiography of paranasal sinuses excluded sinusitis.

**Figure 3:**
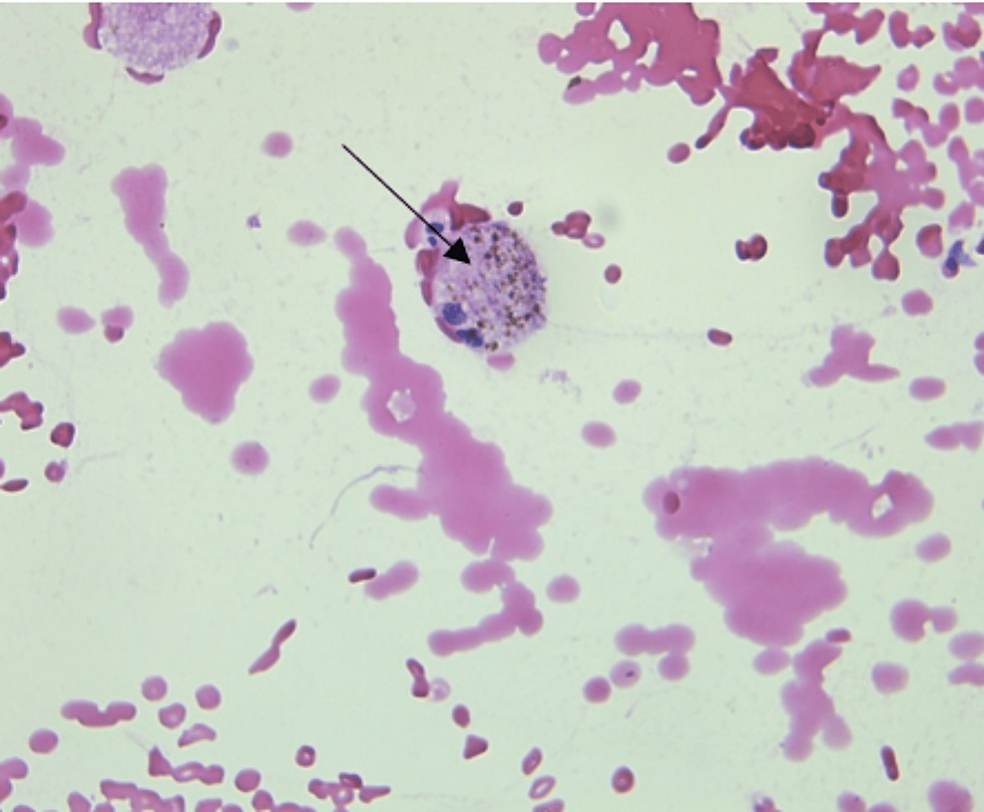
Histopathology of bronchoalveolar lavage fluid showing hemosiderin-laden macrophages (black arrow)

After day 1 of hospitalization, the patient developed respiratory distress with falling SpO_2_ (84%) for which she was shifted to the intensive care unit and started on oxygen support. Her renal function deteriorated on day 2, and she was initiated on dialysis due to hyperkalemia and uremia (urea, 134 mg/dL; serum creatinine, 8.8 mg/dL; and potassium, 6.0 mEq/dL). Bronchoalveolar lavage (BAL) was done. BAL fluid analysis showed hemosiderin-laden macrophages in a fibrin-hemorrhagic background (Figure 3), a moderate number of Gram-negative bacilli, and negative results for malignant cells and fungal elements. BAL fluid culture showed no growth. On further examination, she was found p-ANCA (myeloperoxidase, or MPO) positive with a titer of 63.91 RU/mL, and c-ANCA was negative with a titer of 4.87 RU/mL. Antiglomerular basement membrane (anti-GBM) and double-stranded DNA (dsDNA) antibodies were negative. Her laboratory values are summarized in Table 1.

**Table 1:** Laboratory profile of our patient on admission AST, aspartate aminotransferase; ALT, alanine aminotransferase; GBM, glomerular basement membrane; IgM, immunoglobulin M; IFA, indirect fluorescent antibody; ANA, antinuclear antibody; c-ANCA, cytoplasmic-ANCA; p-ANCA, perinuclear-ANCA; dsDNA, double-stranded DNA; BAL, bronchoalveolar lavage; aPTT, activated partial thromboplastin time.

Laboratory Test	Patient Value	Reference Range
Hemoglobin (gm/dL)	5.5	13-17
Total leukocyte count (cells/cumm)	10,070	4,000-10,000
Platelet count (cells/cumm)	127,000	150,000-410,000
Urea (mg/dL)	128	16-40
Serum creatinine (mg/dL)	7.95	0.90-1.30
Sodium (mEq/L)	137.8	136-145
Potassium (mEq/L)	5.5	3.5-5.5
Total bilirubin (mg/dL)	0.315	0.20-1.10
Direct bilirubin (mg/dL)	0.144	<0.20
AST (U/L)	20	19-48
ALT (U/L)	16	13-40
Albumin (g/dL)	2.98	2.8-4.4
Total protein (g/dL)	5.9	6.2-8.1
Anti-GBM IgM (IFA)	Negative	Titre 1:10
ANA (IFA)	Negative	Dilution 1:100
c-ANCA (PR3) RU/mL	4.87	<20
p-ANCA (MPO) RU/mL	63.91	<20
dsDNA (IU/mL)	25.76	<100
Blood culture	Sterile	
BAL culture	Sterile	
aPTT (seconds)	10.4	10.3

Because of active hemoptysis and hypoxemia, she was started on plasmapheresis and immunosuppression (steroids + cyclophosphamide). Methylprednisolone 1 g/day IV was given for three days followed by oral steroids. She was started on oral cyclophosphamide 1.5 mg/kg body weight.

**Figure 4:**
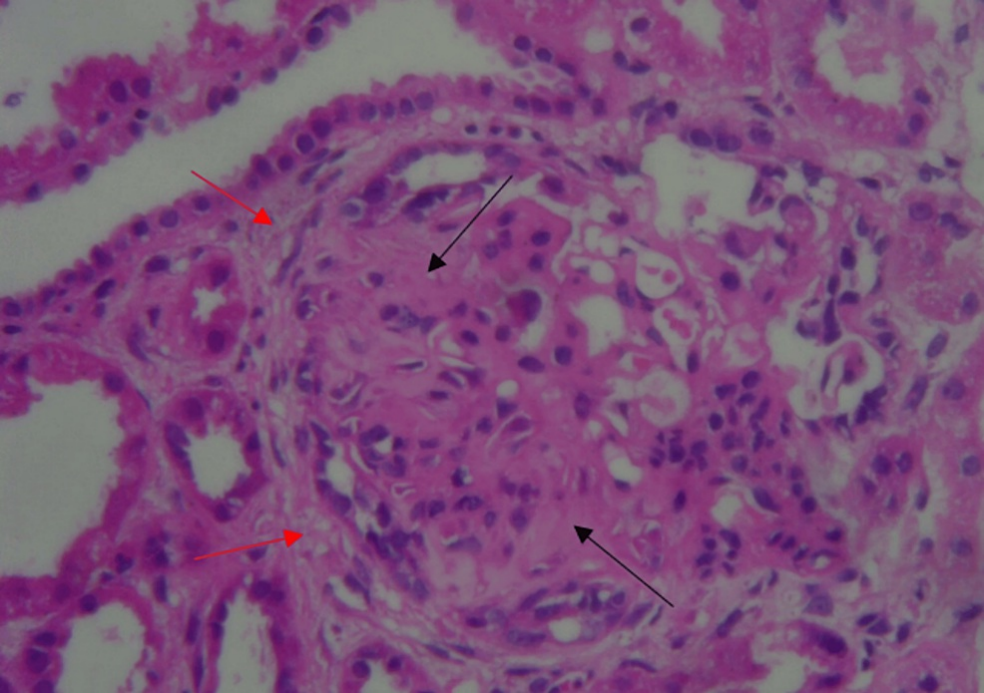
Renal histopathology showing global glomerulosclerosis (black arrows) with fibrous and fibro-cellular crescents (red arrows)

**Figure 5:**
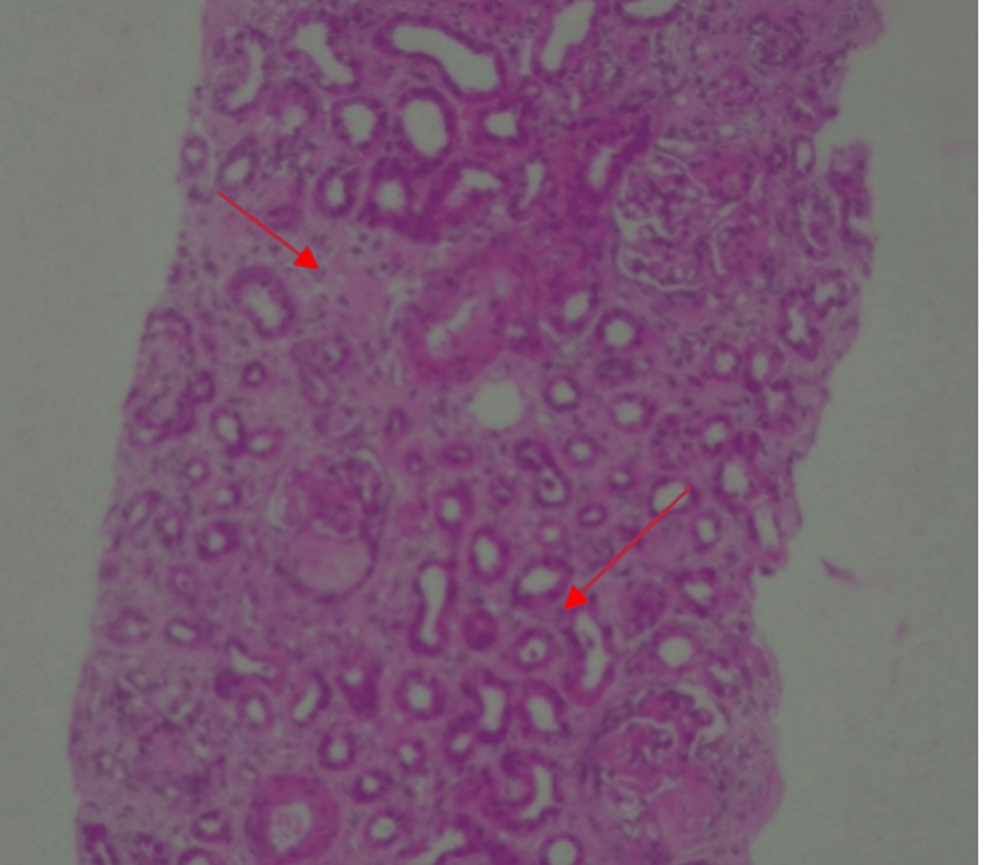
Renal histopathology showing interstitial fibrosis and tubular atrophy (red arrows)

**Figure 6:**
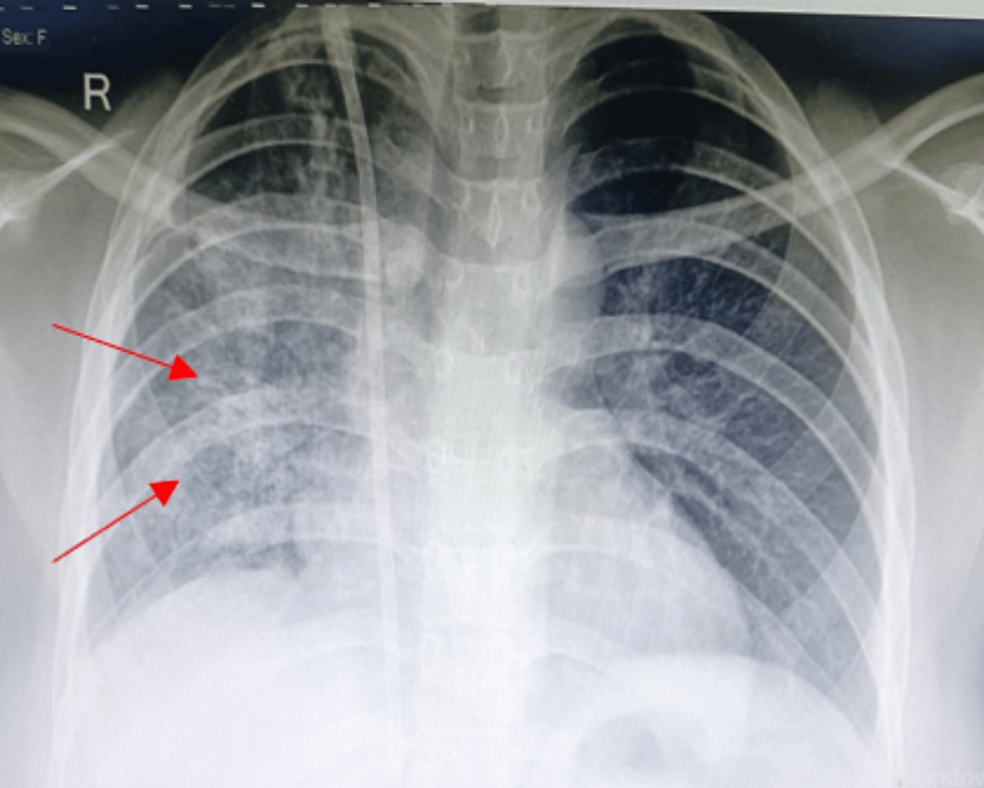
Chest X-ray showing resolving lung infiltrates after treatment (red arrows)

Renal biopsy was done after stabilization of clinical condition, which showed 16 glomeruli out of which 12 were globally sclerosed and another four showed sclerosis with fibrous and fibro-cellular crescents (Figure 4). Interstitial fibrosis and tubular atrophy were present in 50% of tubules (Figure 5). Small blood vessels showed endothelitis. Immunofluorescence was inconclusive as most of the glomeruli were sclerosed. After seven days in intensive care, she maintained a saturation of 98% on room air and was transferred to the ward. She improved clinically with no further hemoptysis and breathlessness. Serial chest X-ray showed a gradual decrease in lung infiltration (Figure 6). Her cyclophosphamide was withheld at discharge (day 21) due to leukopenia. She was discharged in a hemodynamically stable condition on oral steroids with instructions to follow up for completion of induction therapy with cyclophosphamide and later initiation of maintenance therapy.

## Discussion

AAV is an umbrella term for a group of vasculitides that include granulomatosis with polyangiitis, MPA, and eosinophilic granulomatosis with polyangiitis, which are caused by circulating ANCA leading to inflammation and destruction of small and medium-sized blood vessels [2]. Etiopathogenesis is largely attributed to ANCA; however, other factors such as infections, drugs (hydralazine, minocycline, sulfasalazine, and thionamides), and genetic factors may also play a role [3].

Exposure of neutrophils to inflammatory cytokines leads to surface antigens exposure such as myeloperoxidase (MPO) and proteinase 3 (PR3). ANCA binds to one of these exposed antigens leading to the formation of an antigen-antibody complex. This complex causes neutrophil and alternate complement pathway activation followed by damage to the host vasculature by reacting and crosslinking neutrophils to the endothelial receptors [4]. Such type of inflammation in the pulmonary vessels leads to diffuse alveolar hemorrhage. In our patient, the constellation of p-ANCA (63.91 RU/mL) along with hemoptysis and glomerulonephritis is highly indicative of MPA. As there was no medication history, p-ANCA was attributed to microscopic polyangiitis in our case.

MPA is a subtype of ANCA vasculitis, causing non-granulomatous necrotizing vasculitis of small blood vessels [5]. In our case, hypoxic respiratory failure, strongly positive p-ANCA, biopsy-proven glomerulonephritis without granuloma, and radiographic and bronchoscopic evidence of DAH alluded to the diagnosis of MPA. MPA often presents with a long prodromal phase that includes constitutional symptoms such as fever, malaise, and weight loss as well as arthralgias followed by the development of rapidly progressive renal failure. Glomerulonephritis is essentially a universal finding in MPA [2].

The incidence of pulmonary involvement among patients with MPA has been reported to be 60%-70%. Pathologic capillaritis with DAH is the most common manifestation present in 29%-36% of the patients [1]. Necrosis of alveolar structures, loss of capillary structural integrity, and leaking of red blood cells into the alveolar space are all caused by neutrophilic infiltration of the lung interstitium, leading to DAH [6]. It usually presents as multi-lobar and bilateral lung field involvement; however, focal or unilateral distribution should not exclude diffuse alveolar hemorrhage [7]. DAH increases mortality in patients with MPA; especially in an acute clinical setting, 30% of patients do not survive an episode of DAH [1]. Our patient also presented with unilateral lung infiltration, which was confirmed on BAL, and she responded well to plasma exchange and immunosuppressive medications.

In a study of 112 patients with DAH of various causes, 13 patients (11.2%) exhibited unilateral infiltrates on chest imaging. Of all the patients, 34.8% had immune-related DAH out of which only one patient had unilateral lung infiltrates. However, negative pressure pulmonary edema, some drugs, and infections were also reported to cause unilateral DAH in their study [8]. In another retrospective study conducted by Quadrelli et al. involving 39 patients with DAH, it was reported that ANCA-related vasculitis was the predominant cause (74.3%) of DAH in the patients. Out of these, 5.1% of the patients had unilateral DAH [9].

Right-sided unilateral DAH is usually caused by pulmonary congestion due to cardiac failure; however, it was reported to be immune mediated (microscopic polyangiitis) by Kim et al. [10]. In our case, the patient was diagnosed as MPA with right-sided unilateral DAH as the initial presentation.

DAH is the strongest predictor of early mortality in ANCA vasculitis, which varies between different series, with reported survival ranges between 50% and 82% at one year [11,12]. Hence, prompt diagnosis and treatment are always required.

The mainstay of therapy for patients with alveolar hemorrhage due to MPA includes aggressive immunosuppression and plasma exchange [13,14]. Mechanical ventilation is required in case of severe hypoxemia and respiratory failure to maintain oxygenation. Treatment includes induction regimens to induce remission followed by maintenance regimens to maintain remission and prevent relapse. The standard of care for induction therapy in severe AAV includes a combination of glucocorticoids with either cyclophosphamide or rituximab [2]. Plasma exchange should be considered especially in high-risk patients such as those with DAH-associated hypoxemia [15]. In our case, patient’s lung infiltrates significantly improved with plasmapheresis, high-dose steroids, and cyclophosphamide.

## Conclusions

DAH is a life-threatening condition that requires rapid evaluation and management. Early diagnosis and initiation of effective treatment can lead to better outcomes. Patients presenting with DAH should be evaluated for ANCA vasculitis. Although bilateral DAH is a common presentation, one can misdiagnose this unilateral lung infiltrate as pneumonia, especially in the absence of other associated features. Unilateral alveolar infiltrates should not exclude DAH. It is important to keep a high possibility of DAH and vasculitis in these types of presentations to initiate early treatment to improve survival.
